# Therapists' Perspective on Virtual Reality Training in Patients after Stroke: A Qualitative Study Reporting Focus Group Results from Three Hospitals

**DOI:** 10.1155/2016/6210508

**Published:** 2016-12-12

**Authors:** Ludwig Schmid, Andrea Glässel, Corina Schuster-Amft

**Affiliations:** ^1^Physiotherapy Department, Rehabilitation Centre, KliniK Lengg, Zurich, Switzerland; ^2^Department of Physiotherapy, School of Health Professions, Bern University of Applied Sciences, Burgdorf, Switzerland; ^3^School of Health Professions, Institute of Health Sciences, Zurich University of Applied Sciences, Winterthur, Switzerland; ^4^Research Department, Reha Rheinfelden, Rheinfelden, Switzerland; ^5^Institute for Rehabilitation and Performance Technology, Bern University of Applied Sciences, Burgdorf, Switzerland

## Abstract

*Background*. During the past decade, virtual reality (VR) has become a new component in the treatment of patients after stroke. Therefore aims of the study were (a) to get an insight into experiences and expectations of physiotherapists and occupational therapists in using a VR training system and (b) to investigate relevant facilitators, barriers, and risks for implementing VR training in clinical practice.* Methods*. Three focus groups were conducted with occupational therapists and physiotherapists, specialised in rehabilitation of patients after stroke. All data were audio-recorded and transcribed verbatim. The study was analysed based on a phenomenological approach using qualitative content analysis.* Results*. After code refinements, a total number of 1289 codes emerged out of 1626 statements. Intercoder reliability increased from 53% to 91% until the last focus group. The final coding scheme included categories on a four-level hierarchy: first-level categories are (a) therapists and VR, (b) VR device, (c) patients and VR, and (d) future prospects and potential of VR developments.* Conclusions*. Results indicate that interprofessional collaboration is needed to develop future VR technology and to devise VR implementation strategies in clinical practice. In principal, VR technology devices were seen as supportive for a general health service model.

## 1. Background

**Figure 1:**
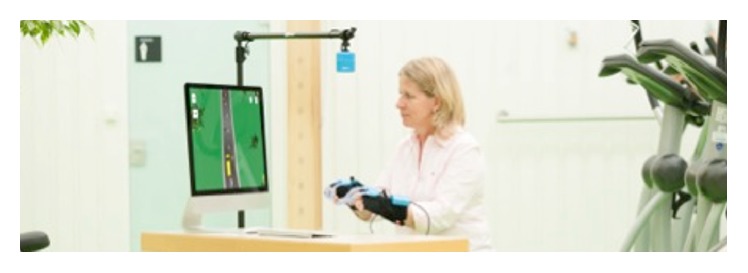
YouGrabber® setup. A model is displayed during virtual reality-based training on the YouGrabber system. The screen shows a game to direct a car on a curvy street with movement of both upper limbs.

Stroke is a frequent cause of livelong disability in adulthood and is one of the most expensive diseases regarding patient-centred care [1]. To reduce the burden of upper limb limitations and to improve patients' outcomes and independence, new treatment concepts have to be developed and effectiveness of patient outcomes has to be investigated, respectively [2]. Virtual reality (VR) is a novel computer technology that was adapted for rehabilitation over the past decade [3]. It is a computer technology that simulates real-life learning while providing augmented feedback and a high intensity of massed practiced tasks [4]. VR can be differentiated into immersive and nonimmersive gaming systems. Immersive systems enable players to move an avatar in a simulated environment. Nonimmersive systems often focus on arm or leg movements in simulated 3D environments [5]. VR provides a safe environment for patients to explore functional capability without interference from their physical or cognitive limitations [6]. As an example of a therapeutic VR system, YouGrabber (YG, YouRehab© Ltd.) will be explored in this study: it is a training system for upper limb training in stroke rehabilitation (Figure 1). It provides training of bimanual reaching and grasping in combination with different game options on a computer or television screen. Patients' movements are captured by two size-adjustable data gloves and infrared arm tracking [7]. As Saposnik and Levin reported in their meta-analysis, there are beneficial effects for upper limb rehabilitation using VR in combination with conventional treatment approaches [8]. Analysed studies evaluated different aspects of VR including number of repetitions and exercise intensity. While rehabilitation targets are functional skills, most of VR implementation is working with simulations that are playful but not directly relevant to patients' daily life [4]. To maximise benefits, the therapeutic application of VR should be compatible with the therapeutic goal setting [9]. Moreover, patients' motivation and attention are important factors stimulating motor relearning after stroke [10].

Hence, patients often describe VR as an opportunity to participate in enjoyable activities bridging environmental or psychological barriers [11]. Patients welcome the opportunity to increase functional independence and to extend therapeutic practice beyond the conventional therapy sessions [12].

However, limited knowledge exists about the therapists' points of view on VR technology in general and their experiences and expectations on VR in stroke rehabilitation in particular.

Research Questions are as follows:

1. What are experiences and expectations of physiotherapists (PT) and occupational therapists (OT) in rehabilitation using a VR training system?
2. What are important facilitators, barriers, and risks for implementing VR training in clinical practice?

The YouGrabber training system (YG, YouRehab© Ltd.) is an example of a therapeutic VR system for upper limb training in stroke rehabilitation (Figure 1). It provides training of bimanual reaching and grasping in combination with different game options on a computer or television screen. Patients' movements are captured by two size-adjustable data gloves and infrared arm tracking [7].

## 2. Methods

### 2.1. Aim, Design, and Data Collection Process

The study aimed to explore the experiences and expectations of PTs and OTs in neurorehabilitation using a VR training system. The focus group method with a purposeful sampling strategy was selected to collect in-depth information about the phenomenon of professional expert interaction [13]. The framework of this qualitative study design was based on a phenomenology approach to explore participants' conceptions and everyday explanations. Phenomenology is a characteristic approach for collecting and analysing lived experiences and personal opinions, in particular in complex interactions as a therapeutic treatment session [14].

Therapists from three different neurological rehabilitation centres in the German speaking part of Switzerland (Reha Rheinfelden, University Hospital Inselspital Bern, Bürgerspital Solothurn) were invited to participate in one focus group session by email or personal communication. In total, nine occupational therapists and physiotherapists specialised in stroke rehabilitation and experienced in working with the YG system were interviewed once only in their work environment in a multicentre setting. Only interviewer and interviewees were present during the focus groups that were recorded by two digital voice recorders. None of the invited therapists refused to participate or withdraw consent. Researchers' and participants' characteristics were reported following the guidelines of reporting qualitative research (COREQ) in Tables 1 and 2 [15].

### 2.2. Participants

OTs and PT had to fulfil the following selection criteria: (1) experience in working with the YG system in stroke rehabilitation with a minimum of sixteen conducted YG therapy sessions with patients after stroke and (2) ability to speak and understand German. No exclusion criteria were defined. Focus groups were conducted between July and December 2013 and chaired by the first author (LS). All focus groups followed a semistructured interview guide (see Table 3). The interview guide was divided into two main question categories: (1) experiences with and expectations on VR training systems and (2) facilitators, barriers, and risks for implementing VR training in clinical practice in stroke rehabilitation. The interview guide was developed by the first author (LS), checked by a second researcher, and pilot tested for meaningfulness and consistency in a single-test interview with an occupational therapist not involved in the study.

The first author (LS) and the last author (CS) did casually know five out of nine participants before study start. For transparency, the moderator (LS) followed a standardised procedure for focus group organising and conducting and for data recording and handling [16]. At the beginning of the focus groups, the moderator explained the process of the group. During the focus group, the moderator had a neutral and intersubjective position and did not intervene in group discussion. The questions of the moderator were just guiding comments or questions for clarification. He did not doubt the content of the answers. Every participant could speak if he/she wished to, but there was no pressure on the participants to respond to every question. Interviewees were not forced to come to a common conclusion on each question. There were no additional questionnaires for the participants. Additionally, during the focus groups, LS recorded aspects of interaction and emotions in field notes. The second author (AG) did not know the participants over the whole study and data analysis period.

### 2.3. Data Preparation

**Figure 2:**
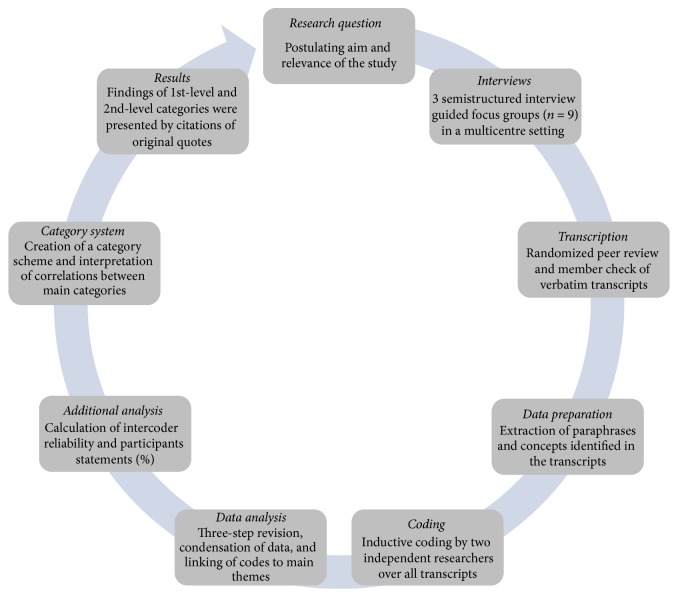
Qualitative research circle applied in the present work: from research question to results.

The data preparation and analysis process is illustrated in Figure 2. All focus group interviews were transcribed verbatim by one researcher (LS) using the software program f5 (Dresing & Pehl GmbH, Marburg, Germany). A simple transcription scheme for qualitative data was used [17]. Swiss German dialect was transferred into standard German. After transcription completion, the text was anonymised and copied to an Excel sheet statement by statement (Microsoft Office 2011, Version 14.3.9, Microsoft Cooperation, USA). After the transfer to Excel, the text was summarised and condensed line by line to enable coding.

For quality assurance and data accuracy of the transcripts, two independent researchers not involved in the study checked. Each of them checked 20% of randomly chosen transcript text based on the guidelines by Elliott et al. [18]. For validity purposes and to avoid misunderstandings, all transcripts were sent to the participants of focus groups to perform a member check. However, findings of the data analysis process were not sent out.

### 2.4. Data Analysis

Focus group transcripts were analysed based on a qualitative content analysis. For the coding process, Excel spreadsheets were used to structure all steps of the content condensation systematically. The number of codes was not calculated. Respectively, the researchers followed a systematic coding process [19] and developed a system of inductive categories. Questions of the moderator were not coded. Each focus group session was transcribed verbatim and data from all three transcripts were analysed independently by two researchers (LS and Andrea Glässel) following a systematic process:

1. After transcription, statements were paraphrased and condensed by LS before starting with the coding process. During the coding process, statements could be assigned to one or more codes, that is, paraphrase: ‘the patients are motivated during the virtual reality training': code allocation “patient motivation.”
2. For calibration, LS and AG independently coded paraphrases line by line based on the coding concept. For peer review, AG additionally coded a randomised part of 20% of all transcript data. Multiple coding was done for cross-checking of coding strategies and interpretation of data by two independent researchers [20].
3. During the coding process, emerging codes and categories were discussed by LS and AG to clarify phrase or code content. Code accordance was given if three or more terms in the code were classified as identical by LS and AG. All remaining codes were marked for discussion. If no agreement was found, a third researcher would have been invited to support code classification, which was not necessary.
4. After independent coding, a formative intercoder reliability of category agreement between LS and AG was calculated.
5. In a refining step, codes from all interviews were collected, condensed, and revised in three steps: (i) removing literal errors, (ii) removing duplicates, and (iii) ordering code sequence. All codes were assigned to a four-level category scheme (see Table 4). For extraordinary codes, a pick-up category was introduced.
6. A native English-speaking researcher checked and verified English translations of the categories.

### 2.5. Ethics, Trustworthiness, and Qualitative Rigour

The study was approved by two ethical committees, the committee of the canton Aargau (study number: 2012/65) and the canton Bern (study number: 220/12), and was conducted in accordance with the Declaration of Helsinki. All participants were informed about the study in oral and written form and gave written informed consent before data collection began.

For critical appraisal, trustworthiness was considered to guarantee the quality of study implementation and data analysis process [21]. Data triangulation by using three different interview settings in three rehabilitation centres confirmed comprehensiveness of the data [22]. The whole analysis process and emerging questions and limitations were documented in a reflexive journal by the moderator who held an insider position as a neutral and intersubjective person [23, 24].

## 3. Results

### 3.1. Codes and Coding Agreement

In total, three focus groups with nine physiotherapists and occupational therapists were conducted. For details, please see Table 2. The duration of the focus groups lasted from 56 to 68 minutes. Based on the transcripts information, a total number of codes of focus group one were summarised in 557 statements resulting in 482 codes, for focus group two 492 statements resulting in 484 codes, and 577 statements resulting in 404 codes for group three. After code refinements by LS, a total number of 1289 codes emerged out of 1626 statements based on all three focus groups (average per group 456 codes). A maximum of six codes were assigned to a participant's statement. The final coding scheme included categories on a four-level hierarchy (see Table 4). First-level categories were (a)* therapists and VR*, (b)* VR device*, (c)* patients and VR,* and (d)* future prospects and developments* of VR.

Intercoder reliability was calculated over all three focus groups with 75% of average agreement.

### 3.2. First First-Level Category: Therapists and Virtual Reality

*Therapists and VR* comprises aspects regarding therapists' clinical reasoning with or without VR. Regarding therapists' criteria of patient selection for* VR* training sessions and ideas for VR health service models, therapists' actions and tasks during YG training were discussed. One participant reported: “*I had a certain curiosity but also big scepticism against virtual reality.”* (Th4) Another participant mentioned: “*Yes, I feel particularly a little less challenged as a therapist during YG training.”* (Th2)

*“Well, I guess the way you speak then changes a little and (.) one talks more about technology than about the patient himself.” (Th2) *

Therapists' motivation has an influence on the usage of technology and on therapists' behaviour and participation in clinical practice:

*“I have just realised I am still motivated, too, my pulse rises up, my blood pressure gets hypertensive; I noticed once when I switched on the device that I am effectively in the game although I am not playing along.” (Th5) *

Considering interaction with the patients, another participant mentioned that for* “the whole social interaction with the patient you need the motivation, and this cannot be done finally by a computer. I think this is our role as a therapist.”* (Th6) Therapists'* “active role in VR Training sessions”* (Th5) is based on their therapy understanding and personal requirements:

*“I do not want to play or just toy, I want the patient to benefit when he or she leaves therapy.” (Th4) *

An explicit description of therapists' specific tasks during VR training became clear in the following statement:

*“There are patients where the YG training works, and there are patients where you have to monitor how they act and even regulate, control, intervene, appropriately adapt the setting and recurrently intervene during the performance.” (Th4)*

This experience was supported by another participant who mentioned the following in VR and using the YG system:

*“It is really important to give good instructions, that you select the starting position consciously, and also modify and adapt, and also shape or change the games; and for this [adjustments] you need therapists, who have experience with patients, who know their strengths and weaknesses.” (Th6) *

In comparison to the conventional treatment approach of occupational or physiotherapy in treating patients after stroke,* “…to integrate the whole body is easier in conventional therapy than during the YG training.” (Th6)*

However, in consideration of therapists' actions,* “…it is less the handling that which you have to know but more the analysis with adaption (…).” (Th9)*

The category “health service model” describes aspects related to therapy settings, patient supervision, economic aspects, and suggestions for a meaningful application of VR. One therapist said the following:

*“I think for sure that (...) VR will be established, it (VR training) simple should be cheaper, that would be the context.” (Th5)*

A crucial point for successful and effective VR training is therapists' criteria of patient selection:

* “Well for me it is the question of what is the therapy goal for the patient and will I reach this goal better with the VR device than with other options.” (Th9) *

*“Well, in my opinion, he must not have pain in the arm.” (Th2)*

*“I guess concerning the seating quality, he should be able to sit stable and upright during this period.” (Th2)*

*“And certain (.) upper extremity motor skills are necessary.” (Th3)*

### 3.3. Second First-Level Category: Virtual Reality Device

*VR device* describes therapists' experiences, expected and unexpected expectations of the YG system, and aspects of device evaluation in general. All of these aspects were merged into one topic due to participants' mixed statements of experiences and evaluation. VR device specifications and handling were often described by comparison with other VR systems:

*“Perhaps, shortly after my experiences with other VR devices, I actually was positively surprised that there are less technical problems with the YouGrabber.” (Th3)*

None of the therapists had specific expectations regarding the VR system before it was implemented in clinical practice:

*“I did not have any special expectations; for me it is simply an enrichment of treatment options and possibilities.” (Th9) *

Participants also reported on patients' comments about different* device characteristics *specifically on graphics requirements. For example, one therapist cited a patient's statement:

*“What kind of a weird graphic is that? Where did you dig this out, it dates from last century.” (Th4). *

In addition to the graphics, participants discussed the gaming character of VR system:

*“Yes, the games that are offered on the YG System seem a little immature for adult people.” (B3, BE 258). “I have the feeling that it is not matching up, the games and daily life oriented training.” (Th6)*

The requested upper limb movements by the* games* on the YG system in general were described as multivariant, complex, and applicable. Some games seemed to be more frustrating and flawing than others; some were considered to be oriented on everyday life situations.

A frequent use of the VR system seems necessary to achieve a proper handling of YG:

*“This was a process until the point that I can now say, all exercises on the YG are applicable and adjustable.” (Th4). “And I have the feeling for me as a therapist, a little bit more experience is needed for estimating the level of training.” (Th9) *

Participants discussed their developments in VR device handling: increasing speed in setting adaption, decreasing fear to handle the technical challenges of the system, and more competence and routine on the computer-based system.

### 3.4. Third First-Level Category: Patients and Virtual Reality

*Patients and VR* presents aspects about patients' development, benefits of the YG training, and motivational aspects of VR-based training:

*“Well, in my experience, it has a very motivating effect on the patients.” (Th3)*

Based on data of the study, principles of motor learning strategies including shaping, variation of trained tasks, and “*getting direct feedback*” (Th4) were prominent reference points for patients' motivation and curiosity to train with the VR device.

*“(..) well, I think it matters a lot to get the feedback that you have improved.” (Th4)*

However, patients'* demotivation* seems to be an influencing factor too. If therapists have the feeling that they “*…cannot put on a show for the patients.”* (TH4) due to malfunction of the VR device, feelings of scepticism, nuisance, pressure, or frustration were the consequence:

*“…and frustration often arises in the context when the gloves go off, when the YG system does not work.” (Th4)*

The second-level category* patients' development* comprises five third-level categories including* patients' behaviour* on the VR training system and more* automation and independence in activities of daily life*:

*“On the YG system patients develop. You can increase the level of difficulty, you can integrate more complex movements, and perhaps you can come to higher selectivity in the finger movement, or come from a complete finger clench to bilateral finger movements at any time.” (Th4) *

*Another participant reported:  “I really suppose that it has to do with the YouGrabber that in daily life, patients made improvements in grasping a knife or reaching objects.” (Th5)*

### 3.5. Fourth First-Level Category: Future Prospects and Developments

The most discussed risk of VR application was the gap between virtual reality and reality in the category* future prospects and developments:*

*“And this lacks the haptic feedback, to perceive a weight or the material of an object, the surface, the temperature, similar points, so VR is a little away from reality.” (Th3)*

Not only device-oriented risks but also patient-centred risks were reported:

*“I think it can be quite a frustrating experience for the patient that a performance works in a VR gaming situation, but not in reality.” (Th4)*

*“Well, I believe the language changes a little bit and therapists are talking more about technology than about the patient.” (Th2)*

* “It is as if the goal setting shifts a little bit more to a good outcome in the game and away from preferably good quality of movement.” (Th4)*

In general, VR training sessions are perceived as* “…a great chance in addition to conventional approaches in occupational and physiotherapy.” (Th7)*

*“Because I really have the feeling that it can not replace you as a therapist. Well, that is what we already had discussions about – what are you actually doing there?” (Th9)*

The opportunity for structured self-training and VR training easiness convinced therapists. The second-level category* future development and networking* refers to participants' impressions on challenges and chances of VR in the future. Networking of different players in the development of new VR technology and its dependence on economic aspects in selling this technology was discussed:

* “Well, I am thinking all these VR systems pushing on the health market or in rehabilitation are not critical reflected, at least not in the period of development. And when in that case the systems are still on the rehabilitation market, they want to be sold.” (Th4)*

Future prospects include ideas for VR devices as “…*good instructed home treatments.” *(Th5) and “…*intensified contact and exchange between therapists and engineers.” *(Th4) to develop VR technology to the end-users' needs.

*“I think VR will be established but I rather think that device material has to get much cheaper and especially less fragile; it has to become more robust.” (Th6) *

### 3.6. First Level: Pick-Up Category

A* pick-up category* was introduced for statements and codes that were not directly related to the research questions associated with YouGrabber. The pick-up category includes two second-level categories: (a) a*dditional experiences with other VR systems *including the Nintendo Wii and (b)* ambience *for VR training.

One therapist said the following:* “Yes (...), we also do playful movement therapy with such a Nintendo Wii console. Once, I tried it with a patient. The main content was that the patient showed me how it works.” (Th4)*

### 3.7. Linkage of All Category Levels

**Figure 3:**
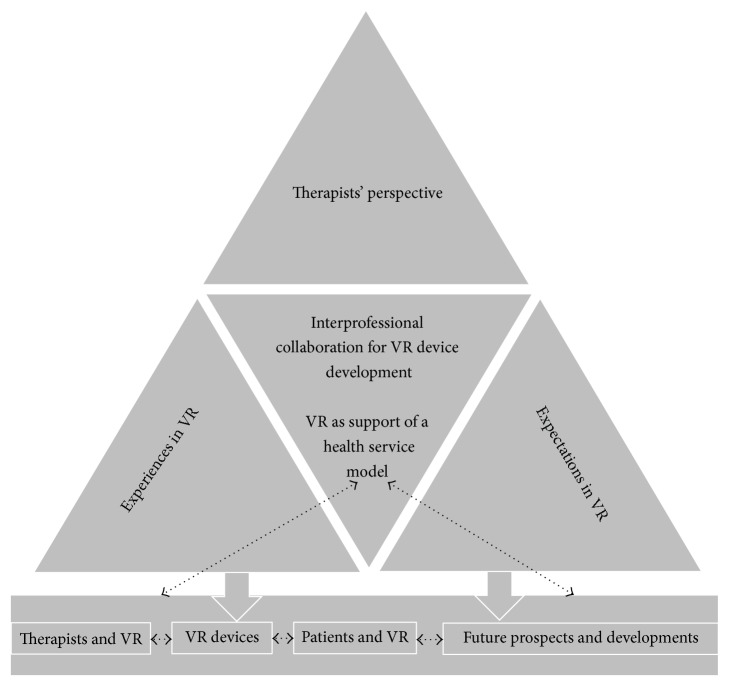
Relationship between research question, first-level categories, and study conclusion. VR: virtual reality.

During the data analysis process, it became obvious that some identified categories were interrelated across hierarchy levels. Based on the provided information on participants' experiences and expectations, a schema of categories and their interrelations emerged (Figure 3).

All categories were connected to the second-level category* health service model*. Different statements independently referred to ideas of patient-centred care, experiences, and opportunities to integrate VR into clinical practice. Therapists' tasks, actions, and clinical reasoning processes for patient selection changed the therapists' role and presented new perspectives in the treatment strategies of stroke rehabilitation.

Patients' needs for increased therapy, continuous motivation, and fast-changing technical opportunities influence the* health service model*. Hence, new technical approaches for patient rehabilitation are needed to provide comprehensive and effective treatments for a growing number of patients in the future.

Motor learning principles of VR-based training including direct feedback or motivational aspects are mutually compatible with technology-based training in neurorehabilitation. Computer-based technology and devices support training variability. The category “*therapists*”* future prospects and developments'* considers aspects named by the therapists including risks and chances of VR application. They connect them with expected improvements in device specifications. Furthermore, therapists claimed an information exchange among researchers, device suppliers, device engineers, therapists, and the users themselves.

## 4. Discussion

The present study provides information on experiences and expectations of physiotherapists and occupational therapist on the training using VR in the rehabilitation of patients after stroke. During three focus group interviews, therapists discussed VR as a complement to conventional treatment approaches and regarding future prospects and developments. Four first-level categories emerged which indicate a positive attitude of the participants regarding VR training in general. Nevertheless, risks and concerns regarding VR technology and the YG system were mentioned.

### 4.1. Therapists and Virtual Reality

In the findings of the first-level category* therapists and VR*, aspects similar to the Technology Acceptance Model of Bertrand and Bouchard (2008) emerged, for example, perceived usefulness of VR training technology [25]. In accordance with the Acceptance Model, therapists in our study did not report anxiety of being replaced in their professional role by computer-based technology.

Furthermore, we identified beneficial effects of VR-based treatments including improvements in motor function which are supported by a literature review from Laver at al. [26]. The identified role of therapists in VR treatment sessions is consistent with new upcoming self-management concepts that emphasise empowerment and self-responsibility in patients after stroke [27].

### 4.2. Virtual Reality Device

*VR device *characteristics were mentioned as being closely related to expected improvements in VR device hardware and software. Therapists required improvements in VR simulation to align better with ADL-relevant tasks. To develop task-oriented VR game characteristics and to obtain hardware that more adaptively handles human movements, participants expressed their desire for an intensive interprofessional collaboration with VR technology developers. The collaboration desire is supported by findings from Tatla et al. [28]. Improved interprofessional cooperation and knowledge exchange between engineers of computer-based devices and therapists as experts of human movements and patient-centred needs in daily living could reduce the gap that currently available technology leaves in end-user satisfaction.

### 4.3. Patients and Virtual Reality

In the present study, participants reported about patients' motivation and awareness during the VR training. The findings of the current study support the results of Finley and Combs who determined direct feedback as an important factor in VR interventions for patients [29]. Our interviewees mentioned motivation, awareness, and direct feedback as beneficial VR training aspects showing the integration of motor learning principles in the VR training device. As a consequence, a discussion about the importance of patient selection by experienced occupational therapists and physiotherapists may arise. We argue that therapeutic skills, qualified patient evaluation, and clinical reasoning processes should be integrated in educational concepts of health professionals as suggested by Edwards et al. [30].

### 4.4. Future Prospects and Developments

Therapists' positive impressions of the easiness of the VR training are in contrast to their apprehension that human movement is more complex and consequently difficult to illustrate in VR. To compensate for the reported lack of sensory input in VR training sessions will be a challenge in future VR development.

Further questions regarding the VR training benefit and its meaningfulness emerged:

*“What benefit of VR training is there for the patient at the end?” (Th4) *

*“Is everything that is technically feasible yet really meaningful?” (Th4).*

### 4.5. Strengths and Limitations of the Study

This is the first qualitative study to explore the perspective of occupational therapists and physiotherapists on VR-based training in patients after stroke. Some limitations appeared.

Saturation of data was achieved because there were no new upcoming categories in the last focus group. Nevertheless, as requested from the COREQ guidelines to confirm extracted findings, data from a fourth focus group would have underlined the criteria of saturation.

Participants were different in the three focus groups regarding distribution of professions and experience. That could have an influence on the variation and depth of responses and the resulting codes based on the therapists' experiences. However, during the time period when the interviews were conducted, therapists in the involved centres only had access to the YG training device.

The choice of a simple transcription scheme to translate the Swiss German dialect into standard German and the translation of codes and quotes into English language could carry a potential risk of losing details. To resolve the issue, a bilingual Swiss German cross-checked transcripts and quotations.

## 5. Conclusions

In summary, main themes of therapists' perspective were related to connections between therapists, patients, device specifications, future prospects, and developments in VR treatments. In general, therapists perceive VR as a useful additional treatment tool complementing conventional methods. Future developments of VR devices will benefit from an interprofessional collaboration between therapists and development engineers. To apply VR as training tools, interprofessional training concepts should be developed with the aim of addressing patients' needs in daily living. Patient motivation and VR training guided by motor learning principles convinced therapists to implement VR training devices in clinical practice. Further research should focus on the effectiveness evaluation of computer-based VR technology, patients' perspective on VR training systems, and how the training success could be transferred into daily life.

## Figures and Tables

**Table 1:** Personal characteristics of all interviewees.

	Researchers	LS	CS	AG
Personal characteristics	Age	31	38	42
	Sex	Male	Female	Female
	Profession	Physiotherapist	Physiotherapist	Physiotherapist
	Credentials	MSc	PhD, MPtSc	PhD, MPH, MSc
	Professional experience (yrs)	6	14	22

Experience VR	Experience YouGrabber®	Yes	Yes	No
	Experience other VR systems	Yes	Yes	No

Experience qualitative research	Experience in chairing interviews (*n*)	2	19	52
	Experience in coding	No	Yes	Yes

Transparency	Transparent assumptions	No	Yes	No
	Transparent personal interest	Yes	Yes	Yes

VR: virtual reality; *n*: number.

**Table 2:** Sociodemographic characteristics of focus group participants.

Part.	ID	Age	Sex	Education	Prof. exp. (yrs)	Patients on YG	Treating YG study patients	Exp. with other VR systems
1	Th1	28	Female	OT	2	55	No	No
2	Th2	37	Female	PT	16	7	Yes	No
3	Th3	38	Female	PT	13	2	Yes	Yes
4	Th4	46	Male	OT	13	60	No	Yes
5	Th5	55	Male	PT	30	3	Yes	No
6	Th6	25	Female	PT	3	3	Yes	No
7	Th7	43	Female	PT	18	6	Yes	Yes
8	Th8	27	Female	PT	5	2	Yes	No
9	Th9	43	Female	OT	19	2	Yes	No

Part.: participants; ID: identification number; Prof. exp.: professional experience; OT: occupational therapist; PT: physiotherapist.

**Table 3:** Interview guide for focus groups.

Sequence	Topic	Number	Question
Introduction		1	What are your general experiences on the YouGrabber system?

Main part	YouGrabber	2	What was your expectation before your first YouGrabber training?
		3	How did you experience the patients on YouGrabber?
		4	What developments did you notice on the patients?
		5	What changes did you make in the handling of YouGrabber?
		6	How did you as a therapist feel during the YouGrabber trainings?

Main part	Virtual reality	7	What facilitators or barriers do you see in the treatment with virtual reality?
		8	What challenges or risks do you see in the treatment with virtual reality?
		9	How do you appraise future possibilities of virtual reality in stroke rehabilitation?

Conclusion		10	Do you have something to add?
		11	What feedback can you give about this interview?

**Table 4:** Category scheme structured on four levels.

First level	Second level	Third level	Fourth level
Therapists and VR	Role of therapists	Interaction Patient management	Active
		Therapeutic outlook	Activity-oriented
			Body function-oriented
		Skills	

Therapists and VR	Therapists' actions and tasks	Approach in OT and PT	Advantages
		Coaching	
		Calibration	
		Documentation	
		Effect of therapy	
		Instruction	
		Patient analysis	
		Patient monitoring	

Therapists and VR	Health service model	Efficiency Patient self-responsibility	
		Therapy setting	Group therapy
			Individual therapy

Therapists and VR	Patient selection	Clinical reasoning	
		Exclusion criteria Stage of rehabilitation	
		Team decision	
		Therapy goal	

VR device	Expected and unexpected expectations and evaluation	Device characteristics	
		No expectations	
		Patient development	
		Patient motivation	
		Risks in VR treatment	
		Therapists' action and tasks	
		Therapist role	

VR device	Device handling and learning effects	General	Negative
			Positive
		Device calibration	
		Experience in handling	
		Fear degradation	
		Handling	Safety in handling
			Speed in handling
			Routine in handling

VR device	Device characteristics	Malfunction	Infrared detection
			Graphics
			Hardware
			Parameter
			Adjustment
			Time delay
		Games	Applicable
			Complex
			Flawed
			Frustrating
			Functional
			Multivariant
			Rare

Patients and VR	Therapy effects on patients	Cognitive function	Awareness
			Concentration
		Emotion	
		Endurance	
		Motor function	Dual task
		Not measurable	
		Pain	
		Vegetative function	

Patients and VR	Patient motivation	Direct feedback	
		Progression	
		Game character	
		Requirement of rehabilitation	
		Influence of media	
		Patient progress	

Patients and VR	Patient demotivation	Patient as test-individual	
		System error	Anxiety
			Frustration
			Nuisance
			Pressure
			Scepticism

Patients and VR	Patient development	Activity of daily life	Transfer in daily life
			No transfer in daily life
			Not measurable
		Automation of movement	
		Behaviour	
		On device	Game
		Independence	

Future prospects and development	Risks and challenges	Contraindications	
		Costs	
		Euphoria	
		Lack of sensory input	
		Misuse	
		Movement quality	
		Transfer into daily life	
		Therapeutic input Mechanisation	
		Workload	
		Self-training	
		Easiness	
		Motor learning principles	Motivation
			Intensity
			Resource-oriented

Future prospects and development	Chances and opportunities in VR		Endurance
			Body function-oriented
			Direct feedback
			Repetition
		Addition to conventional approaches in OT and PT	

Future prospects and development	Future developments and networking	Research	
		Marketing	
		Lobby	
		Supplier	
		Rehabilitation centre	

Future prospects and development	Expected improvements	Games	
		Hardware	
		Software	

Pick-up category	Ambience		
	Virtual reality	Other VR systems	

## Abbreviations

AG: Author Andrea Glässel
COREQ: Consolidated criteria for reporting qualitative research
LS: Author Ludwig Schmid
OT: Occupational therapist
PT: Physiotherapist
VR: Virtual reality
YG: YouGrabber.

## Additional Points

The methodology of this study was developed within the framework of a multicentre randomised controlled trial investigating the effectiveness of the YG training system, Schuster-Amft et al., using mixed methods to evaluate efficacy and user expectations of a virtual reality-based training system for upper limb recovery in patients after stroke: a study protocol for a randomised controlled trial (Trials. 2014; 15(1):350).

## Data Access

The datasets during and/or analysed during the current study are available from the corresponding author on reasonable request. The interview guide is included in the material for publication.
